# Differences in surgical outcomes between cervical goiter and retrosternal goiter: an international, multicentric evaluation

**DOI:** 10.3389/fsurg.2024.1341683

**Published:** 2024-02-06

**Authors:** Federico Cappellacci, Gian Luigi Canu, Leonardo Rossi, Andrea De Palma, Maria Mavromati, Paulina Kuczma, Giacomo Di Filippo, Eleonora Morelli, Marco Stefano Demarchi, Paolo Brazzarola, Gabriele Materazzi, Pietro Giorgio Calò, Fabio Medas, our Mediastinal Goiter Study Collaborative Group, Cristina Soddu

**Affiliations:** ^1^ Department of Surgical Sciences, University of Cagliari, Cagliari, Italy; ^2^ Endocrine Surgery Unit, University Hospital of Pisa, Pisa, Italy; ^3^ Service D'endocrinologie, Diabétologie, Nutrition et éducation du Patient, Hôpitaux Universitaires de Genève, Genève, Switzerland; ^4^ Department of Thoracic and Endocrine Surgery and Faculty of Medicine, University Hospitals of Geneva, Geneva, Switzerland; ^5^ Endocrine Surgery Unit, Department of Surgery and Oncology, University and Hospital Trust of Verona, Verona, Italy

**Keywords:** mediastinal goiter, thyroid surgery, cervicomediastinal goiter, thyroid surgery morbidity, retrosternal goiter

## Abstract

**Introduction:**

Goiter is a common problem in clinical practice, representing a large part of clinical evaluations for thyroid disease. It tends to grow slowly and progressively over several years, eventually occupying the thoracic inlet with its lower portion, defining the situation known as retrosternal goiter. Total thyroidectomy is a standardized procedure that represents the treatment of choice for all retrosternal goiters, but when is performed for such disease, a higher risk of postoperative morbidity is variously reported in the literature. The aims of our study were to compare the perioperative and postoperative outcomes in patients with cervical goiters and retrosternal goiters undergoing total thyroidectomy.

**Methods:**

In our retrospective, multicentric evaluation we included 4,467 patients, divided into two groups based on the presence of retrosternal goiter (group A) or the presence of a classical cervical goiter (group B).

**Results:**

We found statistically significant differences in terms of transient hypoparathyroidism (19.9% in group A vs. 9.4% in group B, *p* < 0.001) and permanent hypoparathyroidism (3.3% in group A vs. 1.6% in group B, *p* = 0.035). We found no differences in terms of transient RNLI between group A and group B, while the occurrence of permanent RLNI was higher in group A compared to group B (1.4% in group A vs. 0.4% in group B, *p* = 0.037). Moreover, no differences in terms of unilateral RLNI were found, while bilateral RLNI rate was higher in group A compared to group B (1.1% in group A vs. 0.1% in group B, *p* = 0.015).

**Discussion:**

Wound infection rate was higher in group A compared to group B (1.4% in group A vs. 0.2% in group B, *p* = 0.006). Based on our data, thyroid surgery for retrosternal goiter represents a challenging procedure even for highly experienced surgeons, with an increased rate of some classical thyroid surgery complications. Referral of these patients to a high-volume center is mandatory. Also, intraoperative nerve monitoring (IONM) usage in these patients is advisable.

## 1 Introduction

Goiter is defined as an abnormal enlargement of the thyroid gland diagnosed at any stage of life. It is a common problem in clinical practice, representing a large part of clinical evaluations for thyroid disease (1, 2). Goiters tend to grow slowly and progressively over several years, and while they are initially diffuse, several factors render them nodular with time (1, 2). The incidence of thyroid goiter strongly depends on the dietary iodine intake, presenting a clear predomination in iodine deficiency areas worldwide (1, 2, 3).

The first description of retrosternal goiter dates back to 1,749 when the term “Retrosternal goiter” was used to qualify an extension of the thyroid gland below the thoracic inlet (4).

Goiter which extends below the thoracic inlet can be classified as primary or secondary, depending on the different blood supply. The primary retrosternal goiter is rare, accounting for less than 1% of retrosternal goiters. It arises from ectopic thyroid tissue in the mediastinum, having its blood supply directly from nonanatomic mediastinal vessel (1, 2, 5–7). The secondary retrosternal goiter arises from an enlarged thyroid gland which has increased its volume and has extended below the thoracic inlet, having its blood supply drawn from the cervical vessels (1, 5, 8–10).

There are many different definitions of retrosternal goiter as a clinical entity in the literature, which consider several criteria that vary from one study to another (6, 7). Depending on the criteria utilized, some practical definitions could be: “a thyroid gland that, on neck examination without being in hyperextension, has a portion that remains permanently retrosternal” (7); “goiter with its lower position permanently remaining below the sternal notch with the neck in hyperextension” (11); or “goiter in which at least 50% is retrosternal” (10). This wide variability of definitions is responsible for the different incidence of retrosternal goiter in the various series, having a reported range of 1%–30% of thyroidectomies (1, 2, 5, 10–12).

Total thyroidectomy (TT) represents the most performed procedure in endocrine surgery; if carried out in high-volume centers, it is associated with low morbidity, mostly represented by recurrent laryngeal injury and post-operative hypoparathyroidism, and virtually no mortality (13–15).

TT is a standardized procedure that represents the treatment of choice for all retrosternal goiters. It could be performed through a cervical or extracervical approach. In most cases, thyroidectomy can be easily conducted through cervical access, but a sternotomy or thoracotomy may be necessary for primary retrosternal goiter and when the gland is predominantly intrathoracic, or when infiltration into surrounding structures is highly suspected at the preoperative assessment (2, 5, 8, 10–12, 16, 17).

However, when total thyroidectomy is performed for retrosternal goiter, a higher risk of postoperative morbidity is variously reported in the literature, mainly represented by postoperative hypoparathyroidism and recurrent laryngeal nerve injury (RLNI) (5, 7, 8, 10–12, 14–18).

To our knowledge, several studies had reported the surgical outcomes of retrosternal goiter to be generically higher than cervical goiter outcomes, while only two large evaluation performed in 2007 by Pieracci et al. and in 2011 by Testini et al. (5, 19), clearly analyzed the difference in terms of surgical morbidity between retrosternal goiter patients and cervical goiter patients.

Several studies report the prevalence of malignancy in retrosternal goiter ranging from 1% to 23%, but only Testini et al. compare rates of malignancy between retrosternal goiter and cervical goiter, finding out an higher prevalence in the first group of patients (2, 5, 8–12, 17, 19).

The aims of our study were to compare the perioperative and postoperative outcomes in patients with cervical goiters and retrosternal goiters undergoing total thyroidectomy. Also, we aimed to assess whether a retrosternal goiter is associated with an increased occurrence of malignancy.

## 2 Materials and methods

This is a multicenter, retrospective, international, observational study that included patients undergoing thyroid surgery from 1st January 2020 to 30th June 2022.

Data were collected from four high-volume thyroid surgery centers in Italy (Cagliari, Pisa and Verona) and Switzerland (Geneva).

Patients submitted to total thyroidectomy for benign and malignant disease were included in this analysis, while our exclusion criteria were as follows: patients with age < 18 years, those who underwent hemithyroidectomy or completion thyroidectomy, simultaneous parathyroidectomy or neck dissection (central and/or lateral), execution of parathyroid autotransplantation, those who underwent surgery with minimally invasive (MIVAT) or remote access such as Robot-Assisted Transaxillary Thyroidectomy (RATT) and TransOral Endoscopic Thyroidectomy Vestibular Approach (TOETVA), and patients with incomplete data.

We included only procedures performed by surgeons with high thyroid surgery experience, that is surgeons performing at least 50 thyroidectomies per year, according to the ESES statement (20).

Parathyroid glands and recurrent laryngeal nerves were systematically searched and identified, as described for the standardized capsular dissection technique.

Energy-based devices (Harmonic Focus—Ethicon, Johnson and Johnson; LigaSure Small Jaw—Medtronic, Covidien Products; and Thunderbeat Open Fine Jaw—Olympus), intraoperative nerve monitoring (IONM) and drain were used based on the preference of the operating surgeon.

The duration of surgery was reported as minutes from skin incision to skin closure.

Retrosternal goiter was defined as a thyroid in which any part of the gland extended below the thoracic inlet with the patient in the surgical position with the neck in hyperextension.

Enrolled patients were divided into two groups: those who had a retrosternal goiter were included in group A, while those who did not have a retrosternal goiter were included in group B.

### 2.1 Endpoints

Our primary endpoint was to estimate the occurrence of post-surgical complications, as listed below: cervical hematoma, recurrent laryngeal nerve injury, post-operative hypoparathyroidism and wound infection.

A 12 month follow up was made to assess the rate of permanent recurrent laryngeal nerve injury and permanent hypoparathyroidism, while cervical hematoma was defined as a post-operative bleeding requiring surgical hemostasis.

Our secondary endpoints were to evaluate whether there was difference in terms of duration of surgery and length of hospitalization between the two groups, and to estimate whether retrosternal goiter was associated with an increased occurrence of malignancy in the final histological evaluation.

### 2.2 Statistical analysis

Calculations were performed with MedCalc® vers. 20.104.

Univariate analysis to compare the two groups was conducted using the chi-square test, or Fisher exact test when appropriate. The presence of a normal distribution of continuous variables was assessed using the D'Agostino-Pearson test. Based on the results of the latter test, Mann–Whitney *U* test was employed for continuous variables, which were expressed as median and interquartile range (IQR).

The results were considered statistically significant for *p*-value < 0.05.

## 3 Results

During the study period, among all centers, a total of 4,467 patients underwent total thyroidectomy for thyroid disease. According to our inclusion criteria, 276 patients (6.2%) presented a retrosternal goiter and were included in Group A, while the remaining 4,191 (93.8%) patients were included in Group B.

Median age was statistically different among our groups, 62 years in Group A vs. 53 years in Group B (*p* < 0.001). Sex female was statistically less frequent in Group A compared to Group B: 182 patients (65.9%) vs. 3,217 patients (76.8%) respectively (*p* < 0.001). Moreover, retrosternal goiter patients in our study presented with higher median BMI than group B patients, 29 Kg/m^2^ vs. 26 Kg/m^2^ (*p* < 0.001) and had more frequently a condition of hyperthyroidism compared to Group B patients: 35.9% (99 patients) vs. 24.7% (1,036 patients) (*p* < 0.001). Interestingly, a histological diagnosis of malignant disease was found more frequently in Group B than in Group A: 83 patients (30.1%) in group A vs. 1,573 (37.5%) patients in Group B (*p* < 0.001). Sample characteristics are summarized in Table 1.

**Table 1:** Sample characteristics.

Item	Group A (*n* = 276)	Group B (*n* = 4,191)	*p*-value
Age, median, (IQR), years	62 (53–71)	53 (42–62)	<0.001
Sex female	182 (65.9%)	3,217 (76.8%)	<0.001
BMI, median, (IQR) kg/m^2^	29 (24.3–35)	26 (23–30)	<0.001
Hyperthyroidism	99 (35.9%)	1,036 (24.7%)	<0.001
Histological diagnosis • Benign diseases • Malignancy	193 (69.9%) 83 (30.1%)	2,618 (62.5%) 1,573 (37.5%)	0.013

Regarding peri-operative characteristics, which are highlighted in Table 2, as foreseeable, we found a higher median operative time in Group A than Group B: 117 min vs. 60 min (*p* < 0.001). Patients in Group A required a median of 2 days of post-operative stays with an IQR of 2–3, while Group B patients had a median post-operative stay of 2 days with an IQR of 1–2 (*p* < 0.001). Notably, there was a much higher usage of IONM in Group A (73.6%) than in Group B (30%, *p* < 0.001). Overall, only 3 patients (1.1% of group A patients) in our study required a complete sternotomy to safely perform the thyroidectomy while 1 patient (0.4% of group A patients) had total thyroidectomy via a combined cervicotomy and Video-Assisted Thoracic Surgery (VATS) approach.

**Table 2:** Perioperative characteristics.

Item	Group A (*n* = 276)	Group B (*n* = 4,191)	*p*-value
Operative time, median, (IQR), minutes	117 (80–147.5)	60 (45–95)	<0.001
Postoperative stay, median, (IQR), days	2 (2–3)	2 (1–2)	<0.001
Use of EBD	261 (94.6%)	2,970 (70.9%)	<0.001
Use of IONM	203 (73.6%)	1,257 (30%)	<0.001
Use of drain	270 (97.8%)	3,802 (90.7%)	<0.001

IONM, intraoperative nerve monitoring; EBD, energy-based device.

Analyzing our primary Endpoints, we found significantly statistical differences in terms of transient hypoparathyroidism (19.9% in group A vs. 9.4% in group B, *p* < 0.001) and permanent hypoparathyroidism (3.3% in group A vs. 1.6% in group B, *p* = 0.035). Regarding the recurrent laryngeal nerve injury rates, we found no differences in terms of transient RNLI between group A and group B, while the occurrence of permanent RLNI was higher in group A compared to group B (1.4% in group A vs. 0.4% in group B, *p* = 0.037). Moreover, no differences in terms of unilateral RLNI were found, while bilateral RLNI rate was higher in group A compared to group B (1.1% in group A vs. 0.1% in group B, *p* = 0.015); all bilateral RLNI occurred in patients who underwent thyroid surgery without IONM.

Wound infection rate was higher in group A compared to group B (1.4% in group A vs. 0.2% in group B, *p* = 0.006). No differences in terms of cervical hematoma requiring surgical hemostasis were found. A summary of our data regarding surgical complications is reported in Table 3.

**Table 3:** Surgical complications.

Item	Group A (*n* = 276)	Group B (*n* = 4,191)	*p*-value
Cervical haematoma	2 (0.7%)	28 (0.7%)	0.708
Transient hypoparathyroidism	55 (19.9%)	396 (9.4%)	<0.001
Permanent hypoparathyroidism	9 (3.3%)	66 (1.6%)	0.035
Unilateral RLNI	11 (4%)	95 (2.3%)	0.069
Bilateral RLNI	3^a^ (1.1%)	6^a^ (0.1%)	0.015
Transient RLNI	10 (3.6%)	84 (2%)	0.069
Permanent RLNI	4 (1.4%)	17 (0.4%)	0.037
Wound infection	4 (1.4%)	9 (0.2%)	0.006

RLNI, recurrent laryngeal nerve injury.

^a^ All bilateral lesions occurred in patients operated without IONM.

## 4 Discussion

Similarly to the previous study by Testini et al. and Pieracci et al., prevalence of retrosternal goiter in our series was 6.2% (5.4% for Testini and 3.4% for Pieracci) (5, 19).

Our study highlights the fact that retrosternal goiter patients represent a slightly different population if compared to other thyroid patients. In our study population, these patients appear to be older and less frequently female when compared with other patients affected by thyroid disease. Retrosternal goiter patients in our study appear to have higher median BMI and presented more frequently a condition of hyperthyroidism compared to Group B patients.

Our data regarding age was comparable to the ones reported by Testini and Pieracci, this difference is probably due to the time needed to the thyroid to increase its volume and overcome the thoracic inlet, thus leading to the older age of these patients (5, 9, 10, 19).

Similar to our study, Pieracci et al. found that retrosternal goiter were less frequently female, however this data was not encountered in the study by Testini et al. (5, 19).

Hyperthyroidism was found to be more frequent in retrosternal goiter patient both by Testini et al. and in our evaluation. This is probably due to the high volume of the thyroid gland in this patient, in turn indicative of a continuous stimulus to glandular growth and hormonal secretion (5).

As predictable, we found that Group A patients presents a higher operative time and had a longer hospital stays if compared to Group B patients. This data are widely reported in the literature and should be taken into account when this procedure is scheduled in these patients (5, 8, 10–12, 17–19).

An invasive thoracic approach was required for 4 patients in our series (1.4% of all retrosternal goiter). This is in line with most of the existing literature, confirming that a cervical approach is feasible in most of these patients (1, 5, 7, 8, 10–12, 17–19).

Regarding our primary endpoint, we found statistical differences in terms of transient and permanent hypoparathyroidism, which appear to be higher in retrosternal goiter patients following TT, as well as permanent RLNI and bilateral RLNI. These results are similar to those reported by Testini et al. and Pieracci et al. in their comparison between cervical goiter and retrosternal goiter: both had found a higher rate of hypoparathyroidism and RLNI in TT performed for retrosternal goiter, although the study by Pieracci et al. do not distinguish between transient and permanent hypoparathyroidism and transient and permanent RLNI (5, 19). Regarding bilateral RLNI, we think that it is important to emphasize that all bilateral lesions occurred in patient submitted to TT without IONM, highlighting the potential of this device, if the two-stage thyroidectomy protocol is adequately followed, to virtually erase the occurrence of this severe complication, as widely demonstrated in the literature (21–26).

We also found differences regarding wound infection rate, that was higher in Group A patients than Group B patients, but this data is in contrast with the other two studies previously mentioned (5, 19).

Several studies report retrosternal goiter as a risk factor for postoperative hematoma following thyroidectomy (27, 28). However, in some series, including a recent study by Canu et al., retrosternal goiter was not found to be related to hemorrhage following thyroid surgery (29). While both Testini and Pieracci found that hematoma following thyroid surgery was mor frequently encountered in retrosternal goiter patients (5, 19), we found no difference in terms of cervical hematoma requiring surgical hemostasis between our groups. However, given the retrospective nature of our study, data regarding hematomas not requiring surgical hemostasis were unavailable; therefore, it is possible that this difference between our study and the others is simply explainable as lack of data in our series. However, it should be noted that we have not observed differences in this sense, given that cervical hematoma requiring surgical hemostasis is in any case the most dangerous occurrence of this type of surgery.

Notably, our study presents an important difference compared to the previous study by Testini et al. (5), in which the authors had found a higher incidence of malignancy in retrosternal goiter than in non-retrosternal goiter, while our data are the opposite, with malignancy to be less frequent in retrosternal goiter patients. In our opinion this difference could be explained by the different time range of the two studies: Testini et al. included cases from 1999 to 2008, while our study included a newer series from 2020 to 2022. We believe that this finding could be explained by an improvement in diagnostic accuracy, especially due to the widespread use of ultrasound, which over the years has led to the early identification of patients at risk of developing a malignant thyroid tumor, therefore intercepting these patients before the gland has time to increase its volume and develop mediastinal extension.

Our study has some limitations. First, its retrospective and multicentric nature, thus leading to a possible different management of some minor aspects of these patients in the various centers (for example IONM usage), although we would like to point out that all patients were operated on by expert surgeons with a high volume of thyroidectomies performed every year. Secondly, it is important to highlight that in the literature there are numerous definitions for the retrosternal goiter, we believe that the one we used to be the most reliable, given the ease of its use in clinical practice, although it is possible that these difference in the retrosternal goiter definitions could be the cause of the variability of data in the literature.

In conclusion, Total thyroidectomy for retrosternal goiter represents a challenging procedure even for highly experienced surgeons. Many classical thyroid surgery complications, such as hypoparathyroidism, both transient and permanent, and permanent RLNI, are more frequently encountered in these patients. Referral of these patients to a high-volume center is mandatory.

Moreover, given our data regarding bilateral RNLI, we think that utilizing IONM in retrosternal goiter patients is advisable.

## Data Availability

The raw data supporting the conclusions of this article will be made available by the authors, without undue reservation.

## Mediastinal goiter study collaborative group

Cristina Soddu, Francesco Casti, Miriam Biancu, Silvia Puddu, Francesca Morinello, Giovanni Lazzari, Dorin Serbusca, Bernard Gjeloshi, Mariangela Caradonna, Luisa Sacco.
