# Supplementary material for: Differences in surgical outcomes between cervical goiter and retrosternal goiter: an international, multicentric evaluation
Source: Front Surg. 2024 Feb 6;11:1341683. doi: 10.3389/fsurg.2024.1341683 (PMC10876881; doi:10.3389/fsurg.2024.1341683)
Supplement: Supplementary file 1 [file Datasheet1.docx]

Mediastinal Goiter Study Collaborative Group:

Giacomo Anedda^1^, Cristina Soddu^1^, Francesco Casti^1^, Miriam Biancu^1^, Silvia Puddu^1^, Francesca Morinello^1^, Enrico Torluccio^1^, Bernard Gjeloshi^2^, Mariangela Caradonna^2^, Luisa Sacco^2^, Giovanni Lazzari^3^, Dorin Serbusca^3^.

^1^Department of Surgical Sciences, University of Cagliari, Cagliari, Italy

^2^ Endocrine Surgery Unit, University Hospital of Pisa, Pisa, Italy

^3^ Endocrine Surgery Unit, Department of Surgery and Oncology, University and Hospital Trust of Verona, Verona, Italy
